# Variation in Acute Care Rehabilitation and 30-Day Hospital Readmission or Mortality in Adult Patients With Pneumonia

**DOI:** 10.1001/jamanetworkopen.2020.12979

**Published:** 2020-09-04

**Authors:** Janet K. Freburger, Aileen Chou, Tracey Euloth, Beth Matcho

**Affiliations:** 1 Department of Physical Therapy, School of Health and Rehabilitation Science, University of Pittsburgh, Pittsburgh, Pennsylvania; 2 University of Pittsburgh Medical Center Rehabilitation Services, Pittsburgh, Pennsylvania

## Abstract

**Importance:**

Pneumonia often leads to functional decline during and after hospitalization and is a leading cause of hospital readmissions. Physical and occupational therapists help improve functional mobility and may be of help in this population.

**Objective:**

To evaluate whether use of physical and occupational therapy in the acute care hospital is associated with 30-day hospital readmission risk or death.

**Design, Setting, and Participants:**

This cohort study included the electronic health records and administrative claims data of 30 746 adults discharged alive with a primary or secondary diagnosis of pneumonia or influenza-related conditions from January 1, 2016, to March 30, 2018. Patients were treated at 12 acute care hospitals in a large health care system in western Pennsylvania. Data for this study were analyzed from September 2019 through March 2020.

**Exposures:**

Number of physical and occupational therapy visits during the acute care stay categorized as none, low (1-3), medium (4-6), or high (>6).

**Main Outcomes and Measures:**

Outcomes were 30-day hospital readmission or death. Generalized linear mixed models were estimated to examine the association of therapy use and outcomes, controlling for patient demographic and clinical characteristics. Subgroup analyses were conducted for patients older than 65 years, for patients with low functional mobility scores, for patients discharged to the community, and for patients discharged to a post–acute care facility (ie, skilled nursing or inpatient rehabilitation facility).

**Results:**

Of 30 746 patients, 15 507 (50.4%) were men, 26 198 (85.2%) were White individuals, and the mean (SD) age was 67.1 (17.4) years. The 30-day readmission rate was 18.4% (5645 patients), the 30-day death rate was 3.7% (1146 patients), and the rate of either outcome was 19.7% (6066 patients). Relative to no therapy visits, the risk of 30-day readmission or death decreased as therapy visits increased (1-3 visits: odds ratio, 0.98; 95% CI, 0.89-1.08; 4-6 visits: odds ratio, 0.89; 95% CI, 0.79-1.01; >6 visits: odds ratio, 0.86; 95% CI, 0.75-0.98). The association was stronger in the subgroup with low functional mobility and in individuals discharged to a community setting.

**Conclusions and Relevance:**

In this study, the number of therapy visits received was inversely associated with the risk of readmission or death. The association was stronger in the subgroups of patients with greater mobility limitations and those discharged to the community.

## Key Points

**Question:**

Is the amount of physical and occupational therapy received by patients with pneumonia associated with 30-day hospital readmission or death?

**Findings:**

In this cohort study of 30 746 patients with pneumonia or influenza-related conditions discharged from 12 acute care hospitals in western Pennsylvania, there was a significant inverse association between the amount of therapy received and the risk of 30-day hospital readmission or death.

**Meaning:**

In this study, the amount of therapy received by patients with pneumonia or influenza-related conditions in the acute care setting was associated with decreases in the risk of 30-day hospital readmission or death.

## Teaser

This cohort study evaluates whether the use of physical and occupational therapy in the acute care hospital is associated with 30-day hospital readmission risk or death.

## Introduction

Pneumonia is a leading cause of morbidity, mortality, and hospitalization in US adults.^1,2^ Nearly 1 million older adults are hospitalized each year for community-acquired pneumonia, and more than one-third die within a year.^3^ Pneumonia is also a common reason for hospital readmission, particularly among older adults, who often experience a functional decline during the index admission.^4,5,6,7,8^ Studies have identified an inverse association between functional status and risk of hospital readmission.^9,10,11,12^ In addition, there is a growing body of literature on the detrimental effects of acute care hospitalization on functional independence.^13,14,15,16^ Loyd et al^14^ reported that 30% of adults aged 65 years and older who were hospitalized in medical-surgical acute care experienced hospital-associated disability, defined as loss of independence in activities of daily living (ADLs) following acute hospitalization.^14^ These findings underscore the importance of hospital-based programs that identify adults at risk of hospital-associated disability and that promote mobility and activity to prevent hospital-associated disability.

Physical and occupational therapists play key roles in promoting mobility and improving functional status in the acute care setting. They also assist with discharge planning by determining the most appropriate postacute setting for patients leaving the hospital with limitations in physical function and ADLs. Finally, they provide patient and family education on rehabilitation care after discharge. While the evidence base for the effectiveness of physical and occupational therapy in the acute care setting is fairly robust for the treatment of conditions such as stroke, hip fracture, and joint replacement,^17,18,19,20,21,22,23^ the value of these services for medical conditions such as pneumonia is less clear. Current guidelines for the treatment of pneumonia primarily focus on diagnostic guidelines and pharmacologic interventions^24^ and do not address early mobility among patients hospitalized with pneumonia.

Limited evidence suggests that early mobilization and rehabilitation decrease morbidity and mortality in patients hospitalized with pneumonia.^17,18,19,20,21,22^ In a retrospective cohort study of adults hospitalized with pneumonia and declining physical function, Kim et al^25^ reported that patients receiving more physical therapy per day (ie, ≥30 minutes) had a lower risk of 30-day readmission. In a systematic review of 4 studies, Larsen et al^26^ found that early mobilization by physical therapists (PTs) decreased length of stay for individuals with pneumonia, but had no association with mortality or hospital readmissions. The authors did acknowledge the limited breadth of their review and the need for further research in this area.

Our study attempts to add to the evidence base on early mobility for patients with pneumonia. Our objective was to determine whether the amount of physical and occupational therapy received by adults hospitalized with pneumonia or influenza-related conditions is associated with the risk of 30-day readmission or death. We hypothesized that the amount of therapy received would be inversely associated with the risk of readmission or death.

## Methods

### Data Sources

We examined electronic health records and administrative claims data from a large health care system in western Pennsylvania. These data were supplemented with US census data to obtain a measure of median household income at the patient zip code and with the Social Security Death Index database to identify individuals who died during follow-up. This study was reviewed and classified as exempt by the University of Pittsburgh’s institutional review board; patient consent was waived by the review board. This study followed the Strengthening the Reporting of Observational Studies in Epidemiology (STROBE) reporting guideline.

### Study Design, Setting, and Participants

We conducted a cohort study examining 2.25 years (January 1, 2016, to March 30, 2018) of electronic health records and administrative claims data from a large health care system in western Pennsylvania. We identified adults aged 18 years or older who were discharged from 1 of 12 acute care hospitals with a primary or secondary diagnosis of pneumonia or influenza-related conditions based on *International Statistical Classification of Diseases and Related Health Problems, Tenth Revision *(*ICD-10*) codes (eTable 1 in the Supplement). We excluded individuals who died during their inpatient stays, were transferred to another hospital (ie, short-term acute, long-term acute, psychiatric, federal), or were discharged to hospice.

### Exposure

We examined billing data to identify the number of PT and occupational therapist (OT) visits during the patient’s inpatient stay. For analytic purposes, we summed the number of therapist visits and created a 4-level categorical variable: no visits, 1 to 3 visits (low), 4 to 6 visits (medium), and more than 6 visits (high). We categorized the number of visits based on the tertile distribution of the data from patients who had at least 1 therapist visit. We also created dichotomous variables to indicate whether only a PT saw the patient, only an OT saw the patient, or both therapist types (PT and OT) saw the patient.

### Outcomes

We created a dichotomous outcome variable to indicate within-system hospital readmission within 30 days of discharge or death within 30 days of discharge with or without an in-system readmission. We chose this composite outcome to address the competing risk of death with hospital readmission.^27^ Death outside the health system may have also been secondary to readmission to another hospital outside the system.

### Covariates

We created several covariates to represent demographic characteristics (ie, age, race, insurance status, and median household income based on zip code of residence) and clinical characteristics (ie, length of stay, intensive care unit use, risk of mortality,^23^ illness severity,^23^ comorbidities, discharge destination, and measures of mobility and activity). The risk of mortality and illness severity measures were derived from the All Patient Refined Diagnosis Related Group (APR-DRG) algorithm. The algorithm uses a set of diagnosis and procedure codes to come up with a DRG as well as severity of illness and risk of mortality modifiers.^28,29^ Severity of illness is defined as the extent to which organ systems lose function or have physiologic decompensation; it is categorized as minor, moderate, major, and extreme.^28,30^ Risk of mortality is a measure of the likelihood of in-hospital mortality based on secondary diagnosis, age, principal diagnosis, and certain procedure codes.^11^ APR-DRGs are calculated for all hospitalizations and have traditionally been used by health systems for cost adjustment, but they are also used by researchers to risk-adjust claims data.^31,32,33,34,35,36,37^ We also calculated the Elixhauser comorbidity index^24^ and created indicator variables for the following diagnoses: arrhythmia, pulmonary circulatory disease, neurological disease, kidney failure, liver disease, cancer, coagulopathy, obesity, and weight loss. Discharge destination was classified as home, home with home health, skilled nursing facility, or inpatient rehabilitation facility. Mobility and activity were measured at the time of hospital admission by nursing staff using the 6-Clicks Activity Measure for Postacute Care (AM-PAC). The AM-PAC 6-Clicks, which has established reliability and validity,^38,39,40^ consists of 6 questions regarding patient mobility (eg, turning over in bed, sitting down and standing up from a chair, walking) and 6 questions regarding patient activity (eg, bathing, dressing, toileting) and is scored on a scale from 6 to 24, with lower scores indicating more difficulty performing the tasks. We created categorical variables for the AM-PAC scores based on their tertile distribution. Complete data variable definitions are provided in eTable 2 in the Supplement.

### Statistical Analysis

We generated descriptive statistics for the patients stratified by the amount of therapy received to compare demographic and clinical characteristics of these subgroups. Acknowledging the nested structure of the data, with patients nested within hospitals, we estimated generalized linear mixed models with a random intercept for hospital to account for the within-hospital correlation. Because the AM-PAC mobility and activity scores were highly correlated (Spearman ρ, 0.93), we excluded the AM-PAC activity data from our analysis. We also excluded the severity of illness measure because it was highly correlated with the risk of mortality measure (Spearman ρ, 0.76) and moderately correlated with the Elixhauser index (Spearman ρ, 0.46). A small amount of data was missing (<5%) for the following measures included in our models: AM-PAC mobility, race, income, and risk of mortality. We imputed mean values for the missing data.

We first estimated a model to examine the association between therapy use and 30-day readmission or death, controlling for patient demographic and clinical characteristics. We also estimated this model for the following subgroups: individuals older than 65 years; individuals with low mobility scores (AM-PAC mobility, <18); individuals discharged to the community; and individuals discharged to a post–acute care facility (ie, skilled nursing facility or inpatient rehabilitation facility). We used χ^2^ tests to assess difference in the point estimates of our exposure categories.

We conducted several sensitivity analyses. We performed the analysis on the subgroup of individuals who survived for the first 30 days after discharge and on the subgroup of individuals who had complete data. We also conducted an analysis with total visits categorized by quartiles. Finally, we conducted a time-to-event analysis using a Cox regression model. Statistical signficance was set at *P* < .05, and all tests were 2-tailed. All analyses were conducted using Stata version 16 (StataCorp).

## Results

**Figure 1.**
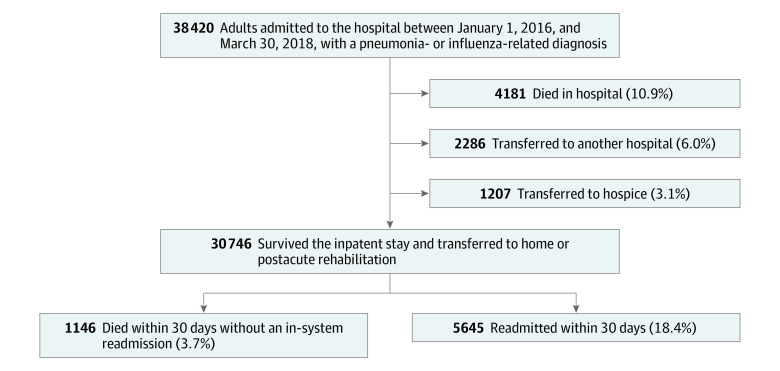
Cohort Selection

Our sample consisted of 30 746 adults with pneumonia or influenza-related conditions who survived their inpatient stay (Figure 1). Rates of discharges per hospital ranged from 849 to 4639 for the entire study period (median [interquartile range {IQR}], 2488 [1719-3426]), with an annual rate ranging from 377 to 1139 (median [IQR], 1106 [764-1522]). Overall, 15 507 patients (50.4%) were men, 26 198 (85.2%) were White individuals, and the mean (SD) age was 67.1 (17.4) years (Table). A total of 20 917 patients (68.0%) had 1 or more therapist visits. Patients who received therapy were older and more likely to be insured by Medicare than those who received no visits (eg, mean [SD] age, 1-3 visits vs no visits: 70.3 [15.6] years vs 58.8 [17.6] years; insured by Medicare, 1-3 visits vs no visits: 5171 of 7901 [65.5%] vs 4751 of 9829 [48.3%]). They also had longer lengths of stay (eg, mean [SD] length of stay, 4-6 visits vs no visits: 8.2 [5.6] days vs 5.3 [5.8] days), were more likely to be in the intensive care unit (eg, 1-3 visits vs no visits: 2100 [26.6%] vs 1638 [16.7%]), had more comorbidities (eg, mean [SD] Elixhauser comorbidity index, 1-3 visits vs no visits: 4.9 [2.2] vs 1.0 [2.2]), had greater illness severity (eg, 1-3 visits vs no visits with extreme illness severity: 2008 [25.4%] vs 1748 [17.8%]) and risk of mortality (eg, 1-3 visits vs no visits extreme risk of mortality: 1634 [20.7%] vs 1128 [11.5%]), and had lower AM-PAC mobility and activity scores (eg, AM-PAC mobility score 6, 1-3 visits vs no visits: 751 [9.5%] vs 762 [7.8%]; AM-PAC activity score 6, 1-3 visits vs no visits: 757 [9.6%] vs 772 [7.9%]). Rates of readmission and death also increased with therapy use (eg, 30-day readmissions: 1-3 visits, 1442 [18.3%]; 4-6 visits: 1120 of 5707 [19.6%]; >6 visits, 1586 of 7309 [21.7%]; death within 30 days: 1-3 visits 310 [3.9%]; 4-6 visits, 242 [4.2%]; >6 visits, 385 [5.3%]).

**Table.** Patient Characteristics and Outcomes by Physical Therapist or Occupational Therapist Use

Characteristic	Patients, No. (%)				
	0 Therapist visits (n = 9829)	1-3 Therapist visits (n = 7901)	4-6 Therapist visits (n = 5707)	>6 Therapist visits (n = 7309)	Total (N = 30 746)
Age, mean (SD), y	58.8 (17.6)	70.3 (15.6)	73 (15.4)	70.3 (16.1)	67.1 (17.4)
Age, y					
18-55	3745 (38.1)	1243 (15.7)	712 (12.5)	1169 (16.0)	6869 (22.3)
56-65	2344 (23.9)	1537 (19.5)	922 (16.2)	1361 (18.6)	6164 (20.1)
66-75	2022 (20.6)	1934 (24.5)	1251 (21.9)	1651 (22.6)	6858 (22.3)
76-85	1171 (11.9)	1752 (22.2)	1446 (25.3)	1781 (24.4)	6150 (20.0)
≥86	547 (5.6)	1435 (18.2)	1376 (24.1)	1347 (18.4)	4705 (15.3)
Men	4993 (50.8)	3903 (49.4)	2791 (48.9)	3820 (52.3)	15 507 (50.4)
Race					
White	7952 (80.9)	6758 (85.5)	5041 (88.3)	6447 (88.2)	26 198 (85.2)
Black	1576 (16.0)	881 (11.2)	483 (8.5)	572 (7.8)	3512 (11.4)
Other	165 (1.7)	120 (1.5)	80 (1.4)	102 (1.4)	467 (1.5)
Missing	136 (1.4)	142 (1.8)	103 (1.8)	188 (2.6)	569 (1.9)
Insurance					
Commercial or other	3091 (31.5)	1786 (22.6)	1376 (24.1)	1905 (26.1)	8158 (26.5)
Medicare	4751 (48.3)	5171 (65.5)	3806 (66.7)	4525 (61.9)	18 253 (59.4)
Medicaid	1987 (20.2)	944 (12.0)	525 (9.2)	879 (12.0)	4335 (14.1)
Income by zip code					
Mean (SD), $	59 326.5 (21 105.3)	58 622 (19 527.1)	60 407.3 (20 974.7)	60 223 (21 322.5)	59 559.4 (20 750.1)
Missing	38 (0.4)	28 (0.4)	15 (0.3)	24 (0.3)	105 (0.3)
Clinical characteristics					
Hospital LOS, mean (SD), d	5.3 (5.8)	5.9 (7.8)	8.2 (5.6)	18.2 (15.9)	9.1 (10.9)
% ICU se	1638 (16.7)	2100 (26.6)	2082 (36.5)	3866 (52.9)	9686 (31.5)
ICU LOS, mean (SD), d	4.2 (6.2)	4.5 (12.5)	5.5 (5.0)	11.5 (13.5)	7.5 (11.4)
Discharge destination					
Home	6979 (71.0)	3296 (41.7)	1135 (19.9)	648 (8.9)	12 058 (39.2)
Home with home health	1983 (20.2)	2611 (33.1)	1807 (31.7)	1902 (26.0)	8303 (27.0)
Skilled nursing facility	819 (8.3)	1763 (22.3)	2232 (39.1)	3572 (48.9)	8386 (27.3)
Inpatient rehabilitation facility	48 (0.5)	231 (2.9)	533 (9.3)	1187 (16.2)	1999 (6.5)
Risk of mortality					
Minor	2262 (23.0)	572 (7.2)	170 (3.0)	95 (1.3)	3099 (10.1)
Moderate	2852 (29.0)	1864 (23.6)	886 (15.5)	606 (8.3)	6208 (20.2)
Major	3587 (36.5)	3831 (48.5)	2816 (49.3)	3007 (41.1)	13 241 (43.1)
Extreme	1128 (11.5)	1634 (20.7)	1835 (32.2)	3601 (49.3)	8198 (26.7)
Missing	0	1 (0.01)	2 (0.03)	6 (0.08)	9 (0.03)
Severity of illness					
Minor	492 (5.0)	154 (2.0)	40 (0.7)	22 (0.3)	708 (2.3)
Moderate	2917 (29.7)	1671 (21.2)	694 (12.2)	387 (5.3)	5669 (18.4)
Major	4674 (47.6)	4068 (51.5)	2847 (49.9)	2566 (35.1)	14 155 (46.0)
Extreme	1746 (17.8)	2008 (25.4)	2126 (37.3)	4334 (59.3)	10 214 (33.2)
Missing	1 (0.01)	1 (0.01)	3 (0.06)	6 (0.07)	11 (0.04)
AM-PAC Mobility					
6, unable to perform any tasks	762 (7.8)	751 (9.5)	602 (10.6)	1041 (14.2)	3156 (10.3)
7-12, major mobility limitations	704 (7.2)	1471 (18.6)	1399 (24.5)	1734 (23.7)	5308 (17.3)
13-18, moderate mobility limitations	925 (9.4)	2107 (26.7)	1796 (31.5)	1868 (25.6)	6696 (21.8)
19-23, minor mobility limitations	1964 (20.0)	1819 (23.0)	1044 (18.3)	887 (12.1)	5714 (18.6)
24, no mobility limitations	5065 (51.5)	1562 (19.8)	712 (12.5)	835 (11.4)	8174 (26.6)
Missing	409 (4.2)	191 (2.4)	154 (2.7)	944 (12.9)	1698 (5.5)
AM-PAC activity					
6, unable to perform any activities	772 (7.9)	757 (9.6)	630 (11.0)	1070 (14.6)	3229 (10.5)
7-12, major activity limitations	482 (4.9)	926 (11.7)	780 (13.7)	1022 (14.0)	3210 (10.4)
13-18, moderate activity limitations	712 (7.2)	1828 (23.1)	1684 (29.5)	1811 (24.8)	6035 (19.6)
19-23, minor activity limitations	1371 (14.0)	1838 (23.3)	1304 (22.9)	1254 (17.2)	5767 (18.8)
24, no activity limitations	6085 (61.9)	2362 (29.9)	1155 (20.2)	1208 (16.5)	10 810 (35.2)
Missing	407 (4.1)	190 (2.4)	154 (2.7)	944 (12.9)	1695 (5.5)
Elixhauser comorbidity index, mean (SD)	4.0 (2.2)	4.9 (2.2)	5.4 (2.3)	6.2 (2.5)	5.0 (2.5)
Outcomes					
Within-system 30-d readmission	1497 (15.2)	1442 (18.3)	1120 (19.6)	1586 (21.7)	5645 (18.4)
Death within 30 d	209 (2.1)	310 (3.9)	242 (4.2)	385 (5.3)	1146 (3.7)
Within-system readmission or death within 30 d	1593 (19.7)	1546 (19.6)	1203 (21.8)	1724 (23.6)	6066 (19.7)

Abbreviations: AM-PAC, Activity Measure for Postacute Care; ICU, intensive care unit; LOS, length of stay.

A total of 12 058 patients (39.2%) were discharged home, 8303 (27.0%) were discharged home with home health, 8386 (27.3%) were discharged to skilled nursing facilities, and 1999 (6.5%) were discharged to inpatient rehabilitation. Overall, 5645 patients (18.4%) had an in-system hospital readmission in 30 days, 1146 patients (3.7%) died within 30 days, and 6066 (19.7%) had an in-system hospital readmission or died within 30 days.

**Figure 2.**
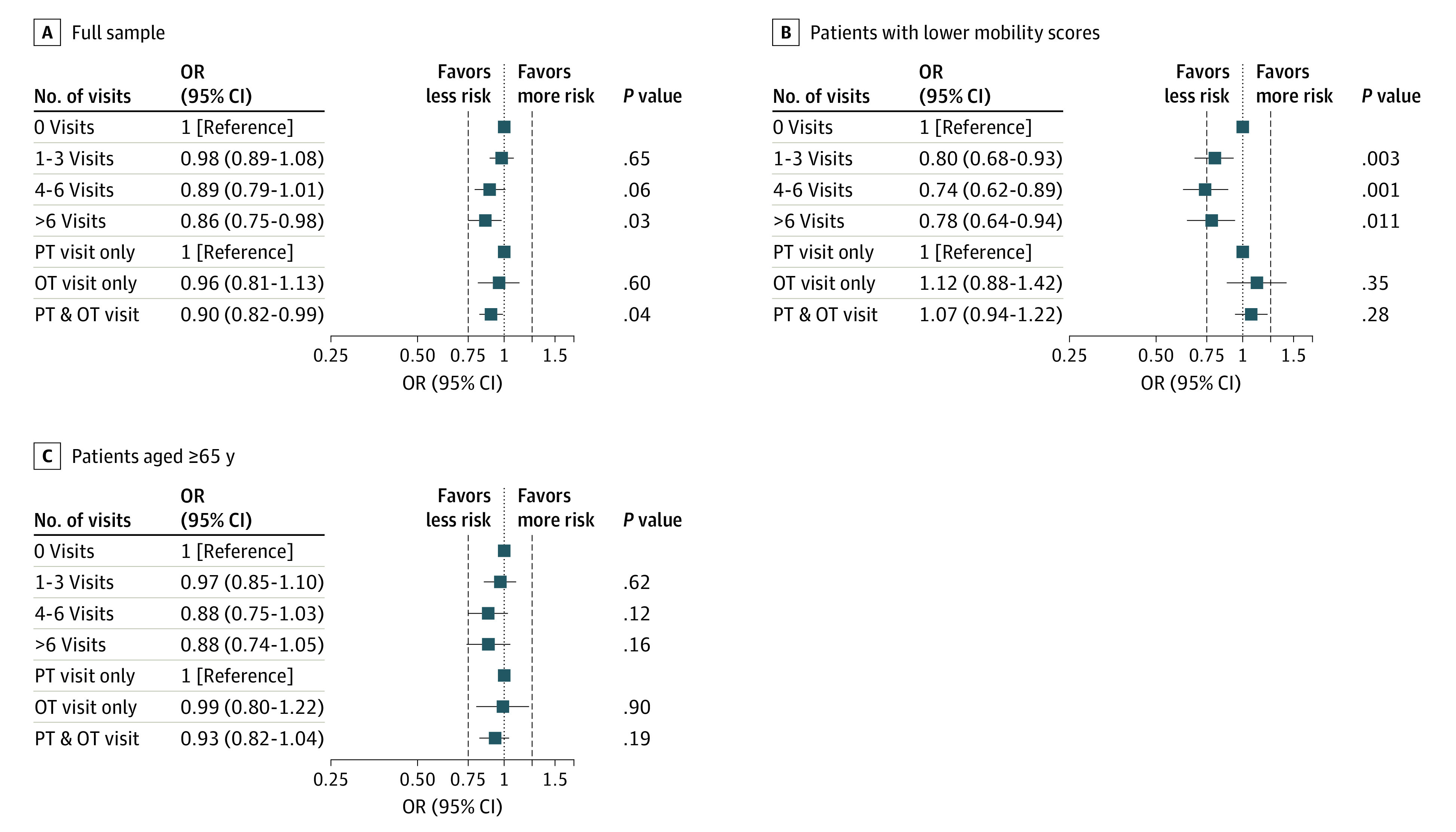
Multilevel Analysis of the Association of Therapy Visits With Risk of 30-Day Hospital Readmission or Death Mixed-effects model with random intercept for hospital, controlling for age; sex; race; insurance; median household income; number of comorbidities; presence of arrhythmia, pulmonary circulatory disease, neurological disease, kidney failure, liver disease, cancer, coagulopathy, obesity, and weight loss; length of stay; intensive care unit use; mortality risk; Activity Measure for Postacute Care mobility scores; and discharge destination. Whiskers represent 95% CIs. OR indicates odds ratio. OT indicates occupational therapist; PT, physical therapist.

Figure 2 presents the results on the association between therapy use and 30-day hospital readmission or death for the full sample. Having 4 or more therapist visits compared with no visits was associated with a decreased risk of 30-day hospital readmission or death (1-3 visits: odds ratio [OR], 0.98; 95% CI, 0.89-1.08; 4-6 visits: OR, 0.89; 95% CI, 0.79-1.01; >6 visits: OR, 0.86; 95% CI, 0.75-0.98). Although the point estimates suggest a slight dose-response pattern, this was not statistically significant. Receiving care from both a physical and occupational therapist decreased the risk of 30-day readmission or death (OR, 0,90; 95% CI, 0.82-0.99). The association of therapy visits was greater when limited to individuals with lower mobility scores, and a dose-response pattern was present when comparing patients who received 1 to 3 visits with those who received 4 to 6 visits (χ^2^ = 11.25; *P* = .003). Being seen by both a physical and occupational therapist was not significant for the lower mobility subgroup. The associations among patients older than 65 years followed a pattern similar to the full sample model, but the odds ratios crossed the null. Our full sample model results are presented in eTable 3 in the Supplement.

**Figure 3.**
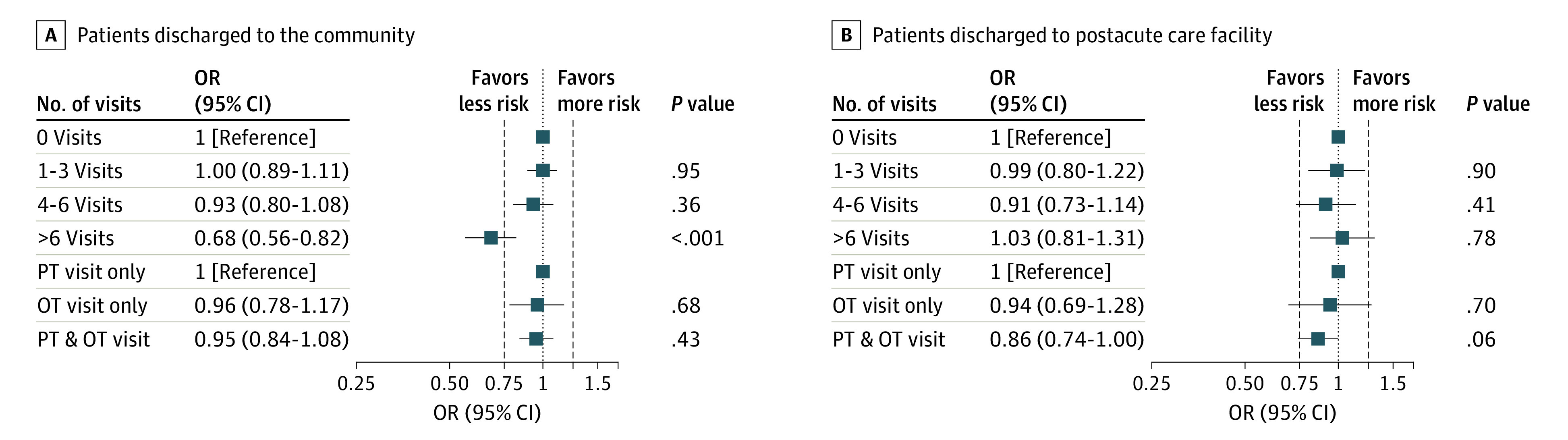
Multilevel Analysis of the Association of Therapy Visits With Risk of 30-Day Hospital Readmission or Death Mixed-effects model with random intercept for hospital, controlling for age; sex; race; insurance; median household income; number of comorbidities; presence of arrhythmia, pulmonary circulatory disease, neurological disease, kidney failure, liver disease, cancer, coagulopathy, obesity, and weight loss; length of stay; intensive care unit use; mortality risk; Activity Measure for Postacute Care mobility scores; and discharge destination. Whiskers represent 95% CIs. OR indicates odds ratio. OT indicates occupational therapist; PT, physical therapist.

Figure 3 presents results on the association between therapy use and hospital readmission or death for individuals discharged to the community or to a post–acute care facility. Having 7 or more therapist visits, compared with no visits, was associated with a decreased risk of hospital readmission or death within 30 days for patients discharged home (odds ratio, 0.68; 95% CI, 0.56-0.82). A dose-response pattern was present when comparing patients who received 4 to 6 visits with those who received more than 6 visits (χ^2^ = 16.2; *P* < .001). Therapy visits were not associated with readmission or death in the subgroup of patients discharged to a post–acute care facility, although being seen by both a PT and OT in the acute care setting decreased risk of readmission or death (OR, 0.86; 95% CI, 0.74-1.00).

### Sensitivity Analyses

When limiting the sample to individuals who survived the first 30 days or individuals with complete data, we found similar results for therapy visits and for seeing both a physical and occupational therapist, although the findings were not significant in some instances (eTable 4 in the Supplement). When creating a 5-level exposure (ie, no therapist visits and therapist visits categorized based on quartile distribution), we saw a more distinctive dose-response pattern (eTable 5 in the Supplement). Results of the Cox regression model for the full sample are presented in eTable 6 in the Supplement. Findings were similar to the logit analysis we conducted. However, the test for proportional hazards was violated in this model.

## Discussion

We examined the association of therapist visits in the acute care setting and the risk of 30-day hospital readmission or death in a cohort of patients with pneumonia or influenza-related conditions. We found that the amount of therapy received was associated with a decreased risk of readmission or death, with some suggestion of a dose-response association. Others have reported a dose-response association between therapy visits and 30-day readmission for patients discharged from acute care for stroke.^28,29,30^ We also found that the associations of therapy with the outcomes were larger for the subgroup of patients with lower mobility (Figure 2) as well as for the subgroup discharged home (Figure 3). These findings make theoretical sense given that individuals with lower mobility are likely to benefit from therapy. Contact with a PT or OT for individuals discharged to the community with functional limitations may also ensure that the patient receives appropriate follow-up care in a timely manner.

Several studies have reported an association between functional decline in the acute care hospital and adverse health outcomes, including hospital readmission.^41,42,43,44^ One mechanism behind our findings is that therapy in the acute care setting decreased the degree of functional decline these patients experienced, thereby reducing readmission risk. Because pneumonia and influenza-related conditions are likely to resolve with appropriate medical management, therapy may be particularly useful in targeting impaired function, a modifiable risk factor for hospital readmission. Hospital-based therapy might also include spillover effects, reducing vulnerability to other acute illnesses.^25^ Finally, contact with a PT and/or OT during the acute care stay may help inform the appropriate discharge destination, which could potentially minimize the risk of adverse outcomes (eg, a patient discharged home with home health that should have been discharged to a skilled nursing facility).

Our findings also suggest that it is the number of therapy visits received, more than the types of therapists seen, that was associated with the risk of readmission or death. In some models, being seen by a PT and OT in the acute care setting decreased the risk of readmission or death compared with only being seen by 1 therapist type. Two therapists working on the patient’s case may facilitate a discharge that is more appropriate for the patient’s needs. Furthermore, because OTs tend to focus more on ADLs, while PTs tend to focus more on mobility (eg, walking, transfers, stairs), there is likely added benefit in addressing both areas.

Our work adds to the evidence on the effectiveness of therapy in the acute care setting for the treatment of pneumonia and influenza-related conditions, medical diagnoses that are less studied than neurologic and orthopedic diagnoses typically treated by therapists. A strength of our study is that we controlled for the degree of difficulty patients had with mobility at hospital admission using AM-PAC scores^38,39,40^ recorded by nursing staff. Unfortunately, we did not have mobility measures at hospital discharge. As others have reported, patients with impaired mobility in our study were more likely to be readmitted, independent of therapist visits (eTable 3 in the Supplement)

Our findings have policy relevance given that pneumonia is among the qualifying diagnoses in the Center for Medicare & Medicaid Services Hospital Readmission Reduction Program, which penalizes hospitals for excess 30-day, unplanned readmissions. Due to the general undersupply of OTs and PTs in US acute care hospitals and high turnover rates,^45,46^ therapy administrators in acute care settings are often challenged to appropriately allocate therapist time. Studies such as this may help inform the prioritization of patients and increase the value add of therapy, which is sometimes viewed by acute care hospital administrators as a cost center only.

### Limitations

This study has several limitations. We examined care in 1 health system in western Pennsylvania, which limits generalizability. We also only captured within-system readmissions. While we were able to capture out-of-system deaths, which may have been preceded by an out-of-system readmission, we did not capture out-of-system readmissions. Our manner of identifying pneumonia patients may also limit generalizability or comparisons with other studies because we included some influenza-related diagnoses. We also only examined all-cause readmissions and not those considered unplanned. Nor did we identify the type of care delivered by therapists. Finally, our study design did not allow for conclusions on causality. Future studies should examine other health systems and geographic populations and explore the amount of time and the specific activities performed during each therapy visit.

## Conclusions

In this study, we examined the association between therapy visits and the risk of 30-day readmission or death in adults hospitalized with a diagnosis of pneumonia or influenza-related conditions. We found that the number of therapy visits received was inversely associated with the risk of readmission or death. This association was greater in the subgroups of patients with lower mobility and patients discharged to the community.

## Notes

Supplement. **eTable 1. ***ICD-10* Diagnosis Codes for Pneumonia **eTable 2. **Variable Definitions and Specifications for Modelling **eTable 3. **Association of Therapy Visits With 30-day Readmission or Death **eTable 4. **Results of Sensitivity Analyses Excluding Individuals Who Died or Had Missing Data **eTable 5. **Sensitivity Analysis With Total Visits Categorized by Quartile Distribution **eTable 6. **Cox Regression Analysis of the Association of Therapy Visits With 30-day Readmission or Death
