# Supplementary material for: Variation in Acute Care Rehabilitation and 30-Day Hospital Readmission or Mortality in Adult Patients With Pneumonia
Source: JAMA Netw Open. 2020 Sep 4;3(9):e2012979. doi: 10.1001/jamanetworkopen.2020.12979 (PMC7489809; doi:10.1001/jamanetworkopen.2020.12979)
Supplement: Supplement. — eTable 1. ICD-10 Diagnosis Codes for Pneumonia eTable 2. Variable Definitions and Specifications for Modelling eTable 3. Association of Therapy Visits With 30-day Readmission or Death eTable 4. Results of Sensitivity Analyses Excluding Individuals Who Died or Had Missing Data eTable 5. Sensitivity Analysis With Total Visits Categorized by Quartile Distribution eTable 6. Cox Regression Analysis of the Association of Therapy Visits With 30-day Readmission or Death [file jamanetwopen-e2012979-s001.pdf]

Supplementary Online Content

Freburger JK, Chou A, Euloth T, Matcho B. Variation in acute care rehabilitation and 30-day hospital readmission or mortality in adult patients with pneumonia. *JAMA Netw Open*. 2020;3(9):e2012979. doi:10.1001/jamanetworkopen.2020.12979

eTable 1. *ICD-10* Diagnosis Codes for Pneumonia

eTable 2. Variable Definitions and Specifications for Modelling

eTable 3. Association of Therapy Visits With 30-day Readmission or Death

eTable 4. Results of Sensitivity Analyses Excluding Individuals Who Died or Had Missing Data

eTable 5. Sensitivity Analysis With Total Visits Categorized by Quartile Distribution

eTable 6. Cox Regression Analysis of the Association of Therapy Visits With 30-day Readmission or Death

This supplementary material has been provided by the authors to give readers additional information about their work.

© 2020 Freburger JK et al. *JAMA Network Open.*

eTable 1. *ICD-10* Diagnosis Codes for Pneumonia

DIAGNOSIS CODE	DIAGNOSIS DESCRIPTION
A481	LEGIONNAIRES' DISEASE
B440	INVASIVE PULMONARY ASPERGILLOSIS
J1082	INFLUENZA DUE TO OTH IDENT INFLUENZA VIRUS W MYOCARDITIS
J129	VIRAL PNEUMONIA, UNSPECIFIED
J159	UNSPECIFIED BACTERIAL PNEUMONIA
J1289	OTHER VIRAL PNEUMONIA
J13	PNEUMONIA DUE TO STREPTOCOCCUS PNEUMONIAE
J154	PNEUMONIA DUE TO OTHER STREPTOCOCCI
J158	PNEUMONIA DUE TO OTHER SPECIFIED BACTERIA
J168	PNEUMONIA DUE TO OTHER SPECIFIED INFECTIOUS ORGANISMS
J180	BRONCHOPNEUMONIA, UNSPECIFIED ORGANISM
J1081	INFLUENZA DUE TO OTH IDENT INFLUENZA VIRUS W ENCEPHALOPATHY
J1100	FLU DUE TO UNIDENTIFIED FLU VIRUS W UNSP TYPE OF PNEUMONIA
J112	INFLUENZA DUE TO UNIDENTIFIED INFLUENZA VIRUS W GI MANIFEST
J1281	PNEUMONIA DUE TO SARS-ASSOCIATED CORONAVIRUS
J14	PNEUMONIA DUE TO HEMOPHILUS INFLUENZAE
J151	PNEUMONIA DUE TO PSEUDOMONAS
B250	CYTOMEGALOVIRAL PNEUMONITIS
J1000	FLU DUE TO OTH IDENT FLU VIRUS W UNSP TYPE OF PNEUMONIA
J101	FLU DUE TO OTH IDENT INFLUENZA VIRUS W OTH RESP MANIFEST
J102	INFLUENZA DUE TO OTH IDENT INFLUENZA VIRUS W GI MANIFEST
J1181	FLU DUE TO UNIDENTIFIED INFLUENZA VIRUS W ENCEPHALOPATHY
J120	ADENOVIRAL PNEUMONIA
J1520	PNEUMONIA DUE TO STAPHYLOCOCCUS, UNSPECIFIED
J156	PNEUMONIA DUE TO OTHER AEROBIC GRAM-NEGATIVE BACTERIA
J181	LOBAR PNEUMONIA, UNSPECIFIED ORGANISM
A3791	WHOOPING COUGH, UNSPECIFIED SPECIES WITH PNEUMONIA
J1083	INFLUENZA DUE TO OTH IDENT INFLUENZA VIRUS W OTITIS MEDIA
J1089	INFLUENZA DUE TO OTH IDENT INFLUENZA VIRUS W OTH MANIFEST
J121	RESPIRATORY SYNCYTIAL VIRUS PNEUMONIA
J122	PARAINFLUENZA VIRUS PNEUMONIA
J123	HUMAN METAPNEUMOVIRUS PNEUMONIA
J150	PNEUMONIA DUE TO KLEBSIELLA PNEUMONIAE
J1108	FLU DUE TO UNIDENTIFIED FLU VIRUS W SPECIFIED PNEUMONIA
J111	FLU DUE TO UNIDENTIFIED INFLUENZA VIRUS W OTH RESP MANIFEST
J153	PNEUMONIA DUE TO STREPTOCOCCUS, GROUP B
J157	PNEUMONIA DUE TO MYCOPLASMA PNEUMONIAE
J17	PNEUMONIA IN DISEASES CLASSIFIED ELSEWHERE
A3781	WHOOPING COUGH DUE TO OTH BORDETELLA SPECIES WITH PNEUMONIA
J1001	FLU DUE TO OTH IDENT FLU VIRUS W SAME OTH IDENT FLU VIRUS PN
J15212	PNEUMONIA DUE TO METHICILLIN RESISTANT STAPHYLOCOCCUS AUREUS
J160	CHLAMYDIAL PNEUMONIA
J690	PNEUMONITIS DUE TO INHALATION OF FOOD AND VOMIT
A3701	WHOOPING COUGH DUE TO BORDETELLA PERTUSSIS WITH PNEUMONIA
J1008	INFLUENZA DUE TO OTH IDENT INFLUENZA VIRUS W OTH PNEUMONIA
J1189	INFLUENZA DUE TO UNIDENTIFIED INFLUENZA VIRUS W OTH MANIFEST
J15211	PNEUMONIA DUE TO METHICILLIN SUSCEP STAPH

© 2020 Freburger JK et al. *JAMA Network Open.*

eTable 1. ***ICD-10*** **Diagnosis Codes for Pneumonia (continued)**

DIAGNOSIS CODE	DIAGNOSIS DESCRIPTION
J1529	PNEUMONIA DUE TO OTHER STAPHYLOCOCCUS
J155	PNEUMONIA DUE TO ESCHERICHIA COLI
J188	OTHER PNEUMONIA, UNSPECIFIED ORGANISM
J189	PNEUMONIA, UNSPECIFIED ORGANISM

© 2020 Freburger JK et al. *JAMA Network Open.*

eTable 2. Variable Definitions and Specifications for Modelling

Variable	Definition	Source
Demographics		
Sex	Categorized as: Male, Female	UPMC Discharge and Inpatient Billing Data
Race	Categorized as: White, Black, Other<br>Missing data replaced with "white"	UPMC Discharge and Inpatient Billing Data
Age	Categorized as: 18 – 55 years, 56 – 65 years, 66 – 75 years, 76 – 85 years, 86 years and older	UPMC Discharge and Inpatient Billing Data
Insurance	Categorized as: Commercial/Other, Medicare, Medicaid	UPMC Discharge and Inpatient Billing Data
Median Household Income	Based on patient ZIP code and categorized based on the quartile distribution; mean value imputed for missing data	Census Data
Clinical		
Length of stay	Categorized as: 0 – 3 days, >3 – 6 days, >6 – 11 days, >11 days based on quartile distribution	UPMC Discharge and Inpatient Billing Data
ICU use	Yes/no: Intensive care unit use during inpatient stay	UPMC Discharge and Inpatient Billing Data
Severity of Illness	APR-DRG classification: categorized as minor, moderate, major, extreme	UPMC Discharge and Inpatient Billing Data
Risk of Mortality	APR-DRG classification: categorized as minor, moderate, major, extreme; mean value imputed for missing data	UPMC Discharge and Inpatient Billing Data
Indicators for comorbidities	Yes/ no: arrhythmia, pulmonary circulatory disease, neurologic disorder, renal failure, liver disease, cancer, coagulopathy, obesity, weight loss	UPMC Discharge and Inpatient Billing Data
Elixhauser comorbidity count	Ranges from 0 – 31; categorized as: 3 or less, 4 – 5, 6 – 7, 8 or more	UPMC Discharge and Inpatient Billing Data
AM-PAC Mobility Score	Categorized by functional mobility limitations: 6 –unable to perform any tasks, 7-12 – major mobility limitations, 13-18 - moderate mobility limitations, 19-23 – minor mobility limitations, 24 – no mobility limitations<br>Moderate mobility limitations imputed for missing data	UPMC Electronic Health Record
Discharge Destination	Categorized as: Home with outpatient services or without home health services, Home with home health services, Skilled nursing facility, Inpatient rehabilitation facility	UPMC Electronic Health Record
Therapy Visits (types)	Categorized as: if billed at least one visit from: Physical Therapy (PT) only, Occupational Therapy (OT) only, Physical Therapy and Occupational Therapy (PT & OT)	UPMC Discharge and Inpatient Billing Data
Therapy Visits (amount)	Categorized as: total number of visits from PT or OT: No visits, 1-3 visits, 4-6 visits, 7 or more visits	UPMC Discharge and Inpatient Billing Data
Outcomes		
Died within 30 days	Yes/no: Died within 30 days with or without an in-system readmission	Social Security Death Index (SSDI)
Within system 30-day Readmission	Yes/no: Readmitted to a UPMC hospital within 30 days	UPMC Discharge and Inpatient Billing Data
Readmitted or died within 30 days	Yes/no: Readmitted within system or died within 30 days	UPMC Discharge and Inpatient Billing Data & SSDI

© 2020 Freburger JK et al. *JAMA Network Open.*

eTable 3. Association of Therapy Visits With 30-day Readmission or Death[1] (N=30,746)

**Variable**			**Odds ratio**	**95% confidence interval**		**p-value**
Therapy Visits:	None		1.00	---	---	---
	1 – 3 visits		0.98	0.89	1.08	0.652
	4 – 6 visits		0.89	0.79	1.01	0.063
	7 or more visits		0.86	0.75	0.98	0.028
Seen by PT only			1.00	---	---	---
Seen by OT only			0.96	0.81	1.13	0.601
Seen by PT and OT			0.90	0.82	0.99	0.037
Male			0.99	0.93	1.05	0.704
Race:	White		1.00	---	---	---
	Black		1.07	0.97	1.18	0.169
	Other		0.83	0.64	1.06	0.136
Age:	18-55 years		1.00	---	---	---
	56-65 years		1.11	1.01	1.23	0.028
	66-75 years		1.09	0.98	1.22	0.098
	76-85 years		1.14	1.02	1.28	0.022
	86 or more years		1.01	0.89	1.14	0.907
Insurance:	Commercial/Other		1.00	---	---	---
	Medicare		1.02	0.95	1.10	0.569
	Medicaid		1.10	0.99	1.22	0.082
Median Household income:		Quartile 1	1.00	---	---	---
		Quartile 2	1.09	1.00	1.19	0.039
		Quartile 3	1.01	0.93	1.10	0.797
		Quartile 4	1.18	1.07	1.29	< 0.001
Length of stay:	0-3 days		1.00	---	---	---
	>3-6 days		1.26	1.14	1.39	< 0.001
	>6-11 days		1.41	1.26	1.57	< 0.001
	>11 days		1.57	1.38	1.78	< 0.001
ICU use			1.05	0.97	1.13	0.225
Mortality risk:	Minor		1.00	---	---	---
	Moderate		1.61	1.38	1.87	< 0.001
	Major		1.75	1.50	2.03	< 0.001
	Extreme		1.83	1.55	2.15	< 0.001
Comorbidities:	Arrhythmia		1.04	0.97	1.11	0.228
	Pulmonary/circulatory		1.07	0.98	1.16	0.143
	Neurologic		0.99	0.92	1.07	0.784
	Renal Failure		1.24	1.15	1.33	< 0.001
	Liver Disease		1.00	0.90	1.12	0.967
	Cancer		1.46	1.34	1.58	< 0.001
	Coagulopathy		1.04	0.95	1.13	0.435
	Obesity		0.89	0.82	0.97	0.006
	Weight Loss		1.07	0.99	1.16	0.072

[1]generalized linear mixed model with random intercept for hospital

© 2020 Freburger JK et al. *JAMA Network Open.*

eTable 3. Association of Therapy Visits With 30-day readmission or death, full model[1] (continued)

Variable		Odds ratio	95% confidence interval		p-value
Comorbidity count:	0 – 3	1.00	---	---	---
	4 – 5	1.12	1.03	1.22	0.012
	6 – 7	1.32	1.20	1.46	< 0.001
	8 or more	1.54	1.35	1.74	< 0.001
AM-PAC mobility score:	6	1.00	---	---	---
	7-12	1.05	0.94	1.17	0.366
	13-18	0.82	0.73	0.91	< 0.001
	19-23	0.94	0.84	1.06	0.291
	24				
Discharge destination:	Home	1.00	---	---	---
	Home health	1.26	1.16	1.37	< 0.001
	Skilled nursing	1.33	1.21	1.45	< 0.001
	Inpatient rehab	1.33	1.16	1.52	< 0.001
Hospital variance		0.01	0.005	0.03	---

[1]generalized linear mixed model with random intercept for hospital

© 2020 Freburger JK et al. *JAMA Network Open.*

eTable 4. Results of Sensitivity Analyses Excluding Individuals Who Died or Had Missing Data[1]

	Original Model (N=30,746)				Excluding People Who Died within 30 days (N=29,600)				Excluding Individuals with Missing Data (N=28,410)			
**Variable**	**Odds ratio**			**p-value**	**Odds ratio**			**p-value**	**Odds ratio**			**p-value**
No Therapy Visits	1.00	---	---	---	1.00	---	---	---	1.00	---	---	---
1 – 3 visits	0.98	0.89	1.08	0.652	1.03	0.93	1.13	0.571	0.94	0.85	1.03	0.172
4 – 6 visits	0.89	0.79	1.01	0.063	0.95	0.84	1.08	0.421	0.84	0.75	0.95	0.007
7 or more visits	0.86	0.75	0.98	0.028	0.92	0.80	1.06	0.240	0.90	0.78	1.04	0.141
Seen by PT only	1.00	---	---	---	1.00	---	---	---	1.00	---	---	---
Seen by OT only	0.96	0.81	1.13	0.601	0.93	0.79	1.11	0.441	1.00	0.84	1.18	0.967
Seen by PT and OT	0.90	0.82	0.99	0.037	0.90	0.82	1.00	0.040	0.97	0.88	1.07	0.573

[1]*Mixed-effects model with random intercept for hospital, controlling for age; sex; race; insurance; median household income; number of comorbidities; presence of the following comorbidities: arrhythmia, pulmonary circulatory disease, neurological disease, renal failure, liver disease, cancer, coagulopathy, obesity, and weight loss; length of stay; intensive care unit use; mortality risk; AM-PAC mobility scores; discharge destination*

© 2020 Freburger JK et al. *JAMA Network Open.*

eTable 5. Sensitivity Analysis With Total Visits Categorized by Quartile Distribution[1] (N=30,746)

Variable	Odds ratio	95% confidence interval		p-value
No Therapy Visits	1.00	---	---	---
1 – 2 visits	0.99	0.90	1.10	0.922
3 – 4 visits	0.91	0.81	1.02	0.117
5 – 8 visits	0.87	0.77	0.99	0.039
9 or more visits	0.76	0.65	0.88	0.000
Seen by PT only	1.00	---	---	---
Seen by OT only	0.94	0.80	1.12	0.507
Seen by PT and OT	0.94	0.85	1.03	0.173

[1]Mixed-effects model with random intercept for hospital, controlling for age; sex; race; insurance; median household income; number of comorbidities; presence of the following comorbidities: arrhythmia, pulmonary circulatory disease, neurological disease, renal failure, liver disease, cancer, coagulopathy, obesity, and weight loss; length of stay; intensive care unit use; mortality risk; AM-PAC mobility scores; discharge destination

© 2020 Freburger JK et al. *JAMA Network Open.*

eTable 6. Cox Regression[1] Analysis of the Association of Therapy Visits With 30-day Readmission or Death (N=30,746)

Variable			Hazard Ratio	95% confidence interval		p-value
Therapy Visits:	None		1.00	---	---	---
	1 – 3 visits		0.98	0.87	1.10	0.683
	4 – 6 visits		0.90	0.80	1.01	0.062
	7 or more visits		0.86	0.77	0.96	0.006
Seen by PT only			1.00	---	---	---
Seen by OT only			0.94	0.83	1.07	0.378
Seen by PT and OT			0.93	0.86	1.01	0.086
Male			0.99	0.94	1.04	0.606
Race:	White		1.00	---	---	---
	Black		1.08	0.99	1.17	0.066
	Other		0.86	0.71	1.04	0.127
Age:	18-55 years		1.00	---	---	---
	56-65 years		1.09	0.99	1.21	0.075
	66-75 years		1.04	0.94	1.14	0.458
	76-85 years		1.07	0.94	1.21	0.323
	86 or more years		0.94	0.83	1.07	0.338
Insurance:	Commercial/Other		1.00	---	---	---
	Medicare		1.09	0.93	1.27	0.284
	Medicaid		1.11	0.95	1.28	0.182
Median Household income:		Quartile 1	1.00	---	---	---
		Quartile 2	1.11	1.01	1.22	0.039
		Quartile 3	1.00	0.89	1.12	0.988
		Quartile 4	1.11	0.95	1.30	0.194
Length of stay:	0-3 days		1.00	---	---	---
	>3-6 days		1.22	1.09	1.36	< 0.001
	>6-11 days		1.36	1.24	1.48	< 0.001
	>11 days		1.50	1.31	1.73	< 0.001
ICU use			1.03	0.96	1.10	0.435
Mortality risk:	Minor		1.00	---	---	---
	Moderate		1.59	1.40	1.82	< 0.001
	Major		1.72	1.49	1.99	< 0.001
	Extreme		1.78	1.56	2.02	< 0.001
Comorbidities:	Arrhythmia		1.04	0.97	1.11	0.304
	Pulmonary/circulatory		1.05	0.99	1.11	0.084
	Neurologic		0.99	0.94	1.04	0.700
	Renal Failure		1.20	1.15	1.26	< 0.001
	Liver Disease		1.00	0.91	1.11	0.942
	Cancer		1.40	1.31	1.49	< 0.001
	Coagulopathy		1.04	0.92	1.17	0.533
	Obesity		0.90	0.84	0.96	0.001
	Weight Loss		1.04	0.95	1.15	0.384

© 2020 Freburger JK et al. *JAMA Network Open.*

eTable 6. Cox Regression Analysis[1] of the Association of Therapy Visits With 30-day Readmission or Death (continued)

Variable		Odds ratio	95% confidence interval		p-value
Comorbidity count:	0 – 3	1.00	---	---	---
	4 – 5	1.10	0.97	1.25	0.140
	6 – 7	1.27	1.09	1.49	0.002
	8 or more	1.43	1.16	1.76	0.001
AM-PAC mobility score:	6	1.00	---	---	---
	7-12	1.04	0.93	1.16	0.461
	13-18	0.85	0.75	0.95	0.006
	19-23	0.96	0.85	1.08	0.524
	24	0.87	0.77	0.99	0.037
Discharge destination:	Home	1.00	---	---	---
	Home health	1.25	1.13	1.40	< 0.001
	Skilled nursing	1.30	1.21	1.40	< 0.001
	Inpatient rehab	1.32	1.19	1.46	< 0.001

[1] clustering on hospital; global test of proportional hazards assumption, chi-square = 233.32, p<0.001.

© 2020 Freburger JK et al. *JAMA Network Open.*
